# Smartwatch-Derived Nocturnal Scratching Metrics Capture Disease Activity and Severity in Pediatric Atopic Dermatitis

**DOI:** 10.3390/jcm15093380

**Published:** 2026-04-28

**Authors:** Fumiko Iwai, Takahiro Nishida, Rei Kanai, Tomoyuki Arima, Takafumi Takase, Shingo Yamada, Mizuho Nagao, Shigeru Suga, Hitoki Kubota, Kazuaki Okamoto, Akihiko Ikoma, Takao Fujisawa

**Affiliations:** 1 Allergy Center, National Hospital Organization (NHO) Mie National Hospital, Tsu 514-0125, Japan; fu_mi_n_1986@yahoo.co.jp (F.I.); t_nishida0930@yahoo.co.jp (T.N.); kanai01.r@gmail.com (R.K.); tomochan1011@gmail.com (T.A.); takatak817@gmail.com (T.T.); shingoru529@yahoo.co.jp (S.Y.); watersail711@gmail.com (M.N.); 2 Department of Child Health and Development, Graduate School of Medicine, Mie University, Tsu 514-0001, Japan; suga.shigeru.ke@mail.hosp.go.jp; 3 Maruho Co., Ltd., 531-0071 Osaka, Japan; kubota_esk@mii.maruho.co.jp (H.K.); okamoto_dix@mii.maruho.co.jp (K.O.); ikoma_eom@mii.maruho.co.jp (A.I.)

**Keywords:** chemokine CCL17, dermatitis, atopic, patient reported outcome measures pediatric dermatology, pruritus, wearable electronic devices

## Abstract

**Background/Objectives**: The itch–scratch cycle is a key driver of exacerbation in atopic dermatitis (AD) and requires objective monitoring, yet patient-reported itch scores are often unreliable in children. This study aimed to evaluate smartwatch-derived nocturnal scratching metrics as digital biomarkers of disease activity and treatment response in pediatric AD. **Methods**: In this prospective observational study, 50 children (median age 9 years) with physician-diagnosed AD wore an Apple Watch with the Itch Tracker application for 5–14 nights during initiation of topical therapy. Three scratch metrics—scratch count rate (SCR), scratch duration ratio (SDR), and scratch burden index (SBI, duration × intensity)—were analyzed. Associations with clinical outcomes [Eczema Area and Severity Index (EASI), Patient-Oriented Eczema Measure (POEM)], serum thymus and activation-regulated chemokine (TARC), and itch numerical rating scale (NRS) were examined. Logistic regression models were evaluated to examine whether these metrics could identify children who achieved clinically meaningful improvement, defined as EASI-50 plus ≥ 4-point POEM reduction. **Results**: All scratch metrics correlated with baseline EASI (r = 0.60–0.64, *p* < 0.001) and serum TARC (r = 0.58–0.60, *p* < 0.001). Reductions in scratching paralleled clinical improvement (r = 0.67–0.71, *p* < 0.0001). Among models, the SBI-based logistic regression demonstrated the best discriminative performance (AUC = 0.78, 95% CI: 0.64–0.92). **Conclusions**: Wearable-derived nocturnal scratching metrics showed moderate but consistent associations with disease severity and short-term improvement. Although predictive capability remains to be established, these metrics may serve as treatment-responsive digital measures. Given the cross-sectional nature of biomarker analyses and other study limitations, further prospective validation is required before clinical application in real-world pediatric AD monitoring.

## 1. Introduction

Pruritus is the hallmark symptom of atopic dermatitis (AD) and a major driver of the itch–scratch cycle, which contributes to disease exacerbation and chronicity [1,2]. Nocturnal scratching not only disrupts sleep quality but may also impair growth, learning, and overall quality of life in children [3,4]. The Harmonizing Outcome Measures for Eczema (HOME) initiative [5] recommends that itch be measured using patient-reported outcome (PRO) instruments such as the numerical rating scale (NRS) [6]. These tools have been validated in adults and are considered clinically useful for assessing disease severity and monitoring treatment response. However, in children, particularly those with early-onset AD, the validity of NRS-based itch assessments remains insufficiently established. Self-reported itch and quality-of-life measures in pediatric populations are inherently challenging to interpret, as children may lack the cognitive ability to accurately describe their symptoms, and proxy reports by caregivers show poor agreement with patient self-reports As a result, subjective itch ratings in pediatric patients can diverge from their actual scratching behavior.

To address this limitation, wearable devices have been developed to objectively measure scratching behavior—most commonly during sleep, when scratching is less consciously suppressed. Several systems, including actigraphy-based wristbands, accelerometer-equipped patches, and smartphone-integrated applications, have been tested and validated for their ability to detect scratching episodes [7]. Nevertheless, most prior studies have focused on technical performance—whether scratching can be detected and with what accuracy—rather than on the clinical validity of these measurements. In particular, the relationship between objectively measured scratching and clinically meaningful outcomes, such as physician-assessed severity, patient-reported symptoms, treatment responsiveness, and biomarkers, has not been fully elucidated, and pediatric-specific evidence is especially scarce.

This study was designed to address these knowledge gaps by evaluating objectively measured nocturnal scratching behavior in children with AD across a broad range of disease severities. Using a commercially available smartwatch (Itch Tracker application on Apple Watch) [8], we quantified multiple scratch-derived metrics and examined their associations with physician-assessed severity, patient-reported outcomes, and a representative serum biomarker. Rather than aiming to predict long-term therapeutic outcomes, we focused on whether aggregated nocturnal scratching metrics reflect contemporaneous disease burden and short-term changes during routine topical treatment. By linking objective digital measures of scratching with clinically relevant endpoints and biological markers, our study aimed to establish their validity and utility as treatment-responsive and interpretable outcome measures in pediatric AD.

## 2. Methods

This observational study is reported in accordance with the STROBE guidelines.

### 2.1. Subjects

Children aged 4 to 15 years with a physician-confirmed diagnosis of AD, based on the criteria of Hanifin and Rajka [9,10], were enrolled. Eligible participants were required to be able to wear an Apple Watch on the wrist during sleep for nocturnal monitoring. Patients who had received systemic treatments such as biologics or Janus kinase (JAK) inhibitors were excluded because the magnitude of therapeutic response with these agents is substantially greater than that typically observed with topical therapy, making combined analysis inappropriate [11,12]. In addition, the number of patients receiving systemic therapy during the study period was insufficient to allow for a separate, adequately powered analysis of this subgroup.

### 2.2. Study Procedures

After enrollment, each patient underwent a monitoring period of 1–2 weeks, with a minimum of 5 nights of recording. Monitoring was restricted to the period between visit 1 (baseline) and visit 2 (end of monitoring) for analysis. Any data recorded after visit 2 were excluded to ensure temporal alignment between predictors and outcomes. During this period, a smartwatch (Apple Watch) equipped with the Itch Tracker application [8] was worn nightly to record scratch-related metrics.

All patients received standard topical treatment consisting of topical corticosteroids and skin care with moisturizers and/or emollients, with or without oral antihistamines, reflecting routine clinical practice. No other systemic treatments were administered. For patients with moderate-to-severe AD, remission induction therapy—a short-term intensive topical regimen aimed at rapidly achieving disease control, as recommended in the Japanese clinical practice guidelines [10]—was provided.

**Figure 1:**
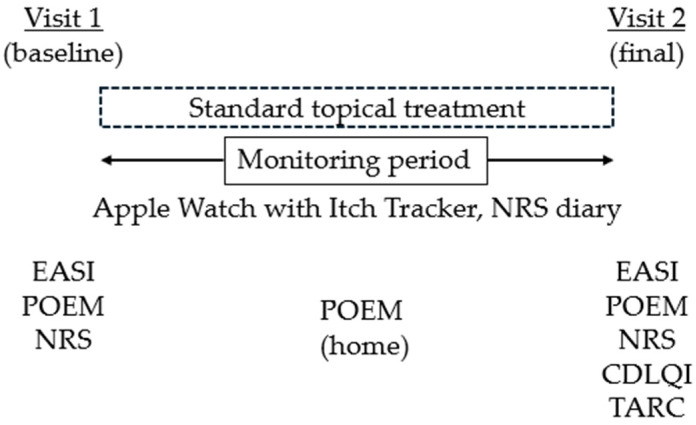
Study design and assessment schedule. Participants underwent two clinic visits (baseline and final), with an interim home-based assessment at the midpoint of the monitoring period. Nocturnal scratching was continuously monitored using the Itch Tracker application, and daytime and nighttime itch were recorded in NRS diaries. Standard topical treatment was maintained throughout the study. Clinical outcomes (EASI, POEM, NRS, CDLQI) and the serum biomarker TARC were assessed at prespecified time points.

Clinical severity was assessed using the Eczema Area and Severity Index (EASI) [13] at baseline (visit 1, hospital), and at the end of the monitoring period (visit 2, hospital). The Patient-Oriented Eczema Measure (POEM) [14] was obtained at baseline (visit 1), at the midpoint of monitoring at home, and at the final assessment (visit 2). The numerical rating scale (NRS) for itch was assessed at visit 1 and visit 2, and also recorded daily at home as daytime and nighttime itch using an itch diary. POEM and NRS were recorded by caregivers, with active input from patients to ensure that their own perceptions and experiences of itch and other symptoms were reflected in the responses. Quality of life was assessed using the Children’s Dermatology Life Quality Index (CDLQI) [15] at visit 2. Blood samples for serum thymus and activation-regulated chemokine (TARC) were obtained only at the final visit (visit 2) [16] (Figure 1), as repeated venipuncture was avoided to minimize burden in pediatric participants.

### 2.3. Smartwatch-Derived Nocturnal Scratch Metrics

Nocturnal scratching behavior was assessed using the Itch Tracker application installed on an Apple Watch (Apple Inc., Cupertino, CA, USA), which utilizes the device’s built-in triaxial accelerometer to capture wrist movements during sleep. Motion signals were analyzed using a proprietary algorithm that identifies repetitive wrist movement patterns consistent with scratching, based on predefined thresholds of acceleration magnitude, rhythmicity, and duration. The algorithm has been validated against synchronized video recordings in adults [8], and captures clinically relevant nocturnal scratching reflecting itch burden. Pediatric-specific validation remains limited; therefore, the present study focused on clinical associations rather than algorithmic validation. Based on these outputs, three scratching metrics were defined in this study:

**Scratch Count Rate (SCR):**

SCR was calculated as $SCR=N_{scratch}/T_{sleep}$ (episodes/hour), where $N_{scratch}$ is the total number of scratch episodes and $T_{sleep}$ is the total sleep time in hours, reflecting the number of scratch episodes normalized by sleep duration.

**Scratch Duration Ratio (SDR):**

SDR was calculated as ***SDR*** = $\sum D_{i}/T_{sleep}\times 100(\%)$, where $D_{i}$ denotes the duration (in seconds) of each detected scratch episode and *T_sleep_* represents the total sleep time in seconds, reflecting the proportion of sleep time spent scratching.

**SBI (scratch burden index):**

SBI was calculated as ***SBI*** = $\sum \left(I_{i}\times D_{i}\right)/T_{sleep}\times 3600$, where $I_{i}$ represents the scratch intensity (m/s^2^) of episode *i*, and $D_{i}$ is its duration in seconds. Scratch intensity was defined as the average peak acceleration during each episode. This composite index integrates both the intensity and duration of scratching to quantify overall nocturnal scratching burden. The unit of SBI is expressed as m/h, derived from acceleration multiplied by time and normalized by sleep duration.

For each metric (SCR, SDR, and SBI), both the mean value across the entire monitoring period and the rate of change (slope) per day were calculated. The mean value was used to capture aggregated nocturnal scratching behavior during the monitoring interval, whereas the slope was estimated by linear regression of nightly values to characterize short-term temporal trends within the same period. Sleep time (*T_sleep_*) was defined as the interval between launching the Itch Tracker application at bedtime and terminating the application upon waking in the morning.

### 2.4. Definition of Clinically Meaningful Improvement (CMI)

In addition to continuous outcomes, clinically meaningful improvement (CMI) was defined according to validated minimal important changes in EASI and POEM, specifically, as the simultaneous achievement of both EASI-50 (≥50% reduction from baseline in EASI) [17] and ≥4-point reduction in POEM [2]. Logistic regression models were constructed using scratch-derived metrics as independent variables, to examine whether these metrics could discriminate participants who achieved CMI. As no external validation was performed, these analyses were considered exploratory and not intended to establish predictive capability.

### 2.5. Statistical Analysis

Baseline characteristics of the recruited subjects were summarized using descriptive analyses. Clinical severity scores were repeatedly assessed during the monitoring period; therefore, paired *t*-tests were used to evaluate changes in EASI between visit 1 and visit 2, whereas mixed-effects models were applied to assess changes in POEM across visit 1, midpoint, and visit 2 since missingness could arise at the home-based midpoint assessments. Mixed-effects models rely on the assumption that data are missing at random (MAR); accordingly, we examined patterns of missingness and found no evidence of informative or nonrandom missingness, supporting the appropriateness of this assumption.

Wearable-derived outputs were treated as continuous measures, whereas clinical scores (EASI and POEM) were analyzed as continuous variables for statistical modeling. The primary aim was to evaluate linear associations between validated clinical scores and scratch-derived metrics. Accordingly, Pearson’s correlation coefficients and simple linear regression were applied to preserve information on effect size and direction. Given the sample size (*n* ≈ 50) and the robustness of Pearson’s method to moderate deviations from normality, this approach was considered appropriate. Serum TARC levels, which typically exhibit a right-skewed distribution with occasional extreme values, were log_10_-transformed a priori to approximate normality and stabilize variance.

To compare the strength of correlations with serum TARC between scratch metrics and clinical severity scores within the same sample, Steiger’s Z test for dependent correlations sharing a common variable was applied [18].

For the outcome of clinically meaningful improvement (defined as EASI-50 plus a ≥4-point reduction in POEM), logistic regression models were fitted to estimate the model-derived probability for each participant. Because the three scratch metrics (SCR, SDR, and SBI) are conceptually related, they were not entered simultaneously into the same model. Instead, each metric was evaluated separately, represented either by its mean value across the monitoring period or by its rate of change (slope) derived from linear regression of nightly values. To address potential collinearity between mean and slope representations of the same metric, variance inflation factors (VIFs) were examined, with values <5 considered acceptable.

Model performance was evaluated in terms of discrimination and calibration. Discrimination was assessed using the area under the receiver operating characteristic curve (AUC) with 95% confidence intervals. Calibration was examined using a calibration plot (observed vs. model-estimated probabilities) and the Brier score. In addition, a calibrated probability curve derived from the logistic model was generated to visualize the relationship between the model-derived score and the model-estimated probability of CMI achievement.

Statistical analyses were performed using JMP Pro (v18), with Steiger’s Z test conducted in Python (v3); graphical visualizations were generated using GraphPad Prism (v10.5).

### 2.6. Ethics Statement

This study was conducted in accordance with the principles of the Declaration of Helsinki. The protocol was reviewed and approved by the Ethics Committee of the National Hospital Organization (NHO) Mie National Hospital (approval number: 2021-69). Written informed consent was obtained from the parents or legal guardians of all participating children prior to enrollment, and assent was obtained from the children when appropriate.

## 3. Results

### 3.1. Clinical Characteristics and Outcomes 

A total of 50 pediatric patients with AD were enrolled, with a median age of 9 years and 60% being boys. The median duration of the monitoring period was 8 nights (range, 5–13 nights). Baseline disease severity, classified according to EASI scores, ranged from mild to severe [13]. At baseline, the median EASI and POEM scores were 11 and 12, respectively. Both significantly decreased to 3.7 and 5 at visit 2 following treatment. Median serum TARC levels at visit 2 were within the normal range overall; however, the distribution was wide, with values reaching up to 9285 pg/mL in some patients. Many participants had comorbid allergic conditions, most commonly food allergy (46%) and allergic rhinitis (72%) (Table 1).

### 3.2. Associations of Mean Scratch Metrics with Clinical Severity

**Figure 2:**
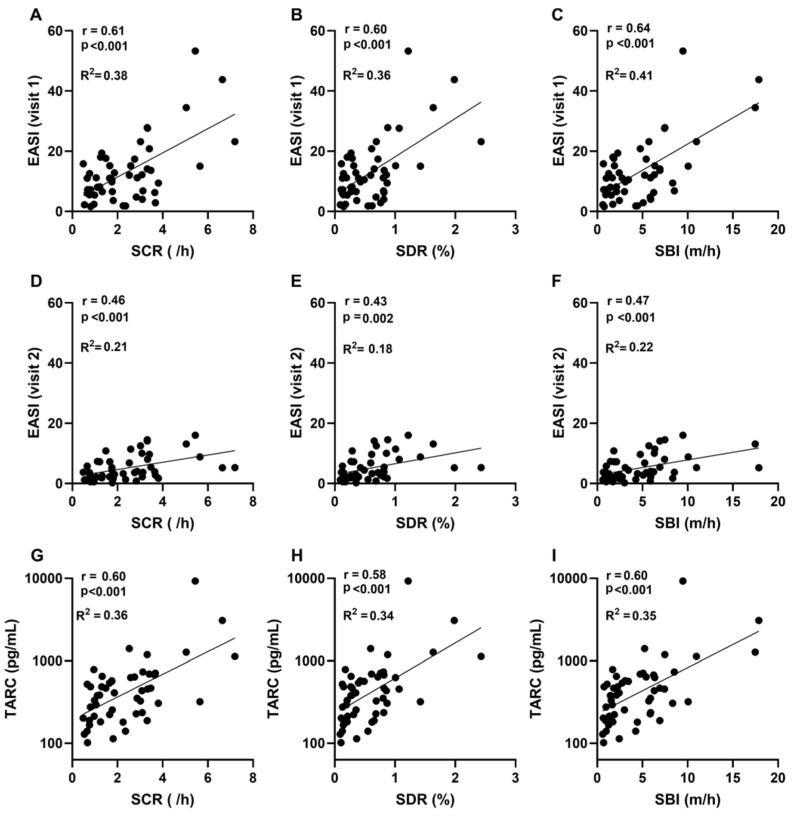
Associations of wearable-derived scratch metrics with disease severity and biomarker levels. (**A**–**C**) Baseline EASI scores assessed at visit 1 in relation to scratch count rate (SCR), scratch duration ratio (SDR), and scratch burden index (SBI) averaged across the entire monitoring period (5–14 nights). (**D**–**F**) Post-treatment EASI scores assessed at visit 2 in relation to the same aggregated scratch metrics (**G**–**I**) Serum TARC levels (visit 2) in relation to the aggregated scratch metrics. Scratch metrics represent mean values across the entire monitoring period (5–14 nights), reflecting aggregated nocturnal scratching behavior as a short-term monitoring indicator rather than day-specific or time-matched fluctuations. Each dot represents an individual participant, and solid lines indicate linear regression fits.

All three mean nocturnal scratch metrics (SCR, SDR, and SBI) correlated significantly with physician-assessed severity (EASI) at both time points: baseline (visit 1) r = 0.60–0.64, all *p* < 0.001 (Figure 2A–C), and post-treatment (visit 2) r = 0.43–0.47, *p* < 0.01 (Figure 2D–F). Corresponding linear fits showed lower explanatory power after treatment.

The scratch metrics also correlated with patient-reported outcomes. Associations with POEM were modest (r ≈ 0.3–0.4, *p* ≤ 0.05) but consistent in direction with those observed for EASI. An exception was noted at the home midpoint, where SDR and SBI showed no significant correlations with POEM (Supplementary Materials Figure S1).

In contrast, no significant correlations were observed between scratch metrics and NRS assessed at visit 1, mean daytime NRS, or visit 2 NRS. Weak associations were observed with mean nighttime NRS (Supplementary Materials Table S1). No correlations were detected between scratch metrics and quality of life as measured by the CDLQI (Supplementary Materials Table S1).

### 3.3. Associations of Mean Scratch Metrics with TARC

In addition to clinical indices, all scratch metrics were significantly correlated with the established biomarker of AD severity, serum TARC measured at visit 2 (r = 0.58–0.60, all *p* < 0.001; Figure 2G–I). Consistent with previous reports, TARC also showed strong correlations with physician-assessed severity (EASI). Interestingly, although TARC was measured at visit 2, its correlation was stronger with baseline EASI (visit 1, r = 0.68) than with follow-up EASI (visit 2, r = 0.45) (Supplementary Materials Table S2). To formally compare these associations, Steiger’s Z test was applied, confirming that the strongest correlation was between TARC and baseline EASI. Notably, correlations between TARC and scratch metrics were significantly stronger than that between TARC and follow-up EASI (all *p* ≤ 0.005). These findings suggest that scratch metrics reflect aspects of disease activity that align with biomarker-based assessments with stronger concordance than EASI measured at the time of biomarker sampling.

### 3.4. NRS Showed No Association with Clinical Indices

In contrast, unlike the objectively measured scratch metrics, the subjective measure of itch intensity, NRS scores (visit 1, mean daytime, and visit 2) showed no significant correlations with either EASI or TARC (Supplementary Materials Figure S2).

### 3.5. Associations of Slopes of Scratch Metrics with Longitudinal Changes in EASI Scores

We next examined whether longitudinal changes in nocturnal scratching behavior were associated with changes in clinical severity (EASI scores) during the monitoring period (Table 2). Slopes of all three scratch metrics (SCR, SDR, and SBI) showed significant positive correlations with the slope of EASI (r = 0.69, 0.71, and 0.67; all *p* < 0.0001). Because EASI scores significantly decreased from baseline (visit 1) to post-treatment (visit 2) (Table 1), these positive correlations indicate that reductions in nocturnal scratching paralleled clinical improvement. Linear regression yielded coefficients of determination (R^2^) ranging from 0.44 to 0.50, suggesting that slopes of scratch metrics moderately reflect longitudinal changes in disease severity.

### 3.6. Logistic Regression Models Examining Associations with Clinical Improvement

Because the mean values of scratch metrics correlated with disease severity and their slopes reflected longitudinal changes, exploratory logistic regression models were developed to examine whether these metrics could distinguish children who achieved clinically meaningful improvement, defined as achieving both EASI-50 and a ≥4-point POEM reduction. Given the very high intercorrelations among mean values of the three scratch metrics (r > 0.90, *p* < 0.001; Supplementary Materials Table S3), mean and slope values of each metric were tested separately in the models.

**Figure 3:**
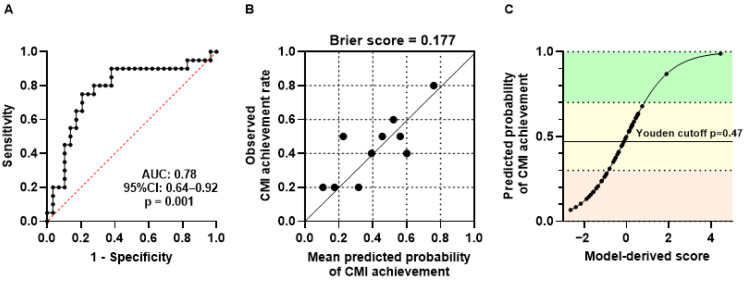
Discriminative performance and model-estimated probability derived from exploratory logistic models examining associations between scratch metrics and clinically meaningful improvement; concurrent achievement of ≥50% reduction in EASI (EASI-50) and a ≥4-point reduction in POEM. (**A**) Receiver operating characteristic (ROC) curve demonstrating discriminative performance of the SBI-based logistic model for identifying children who achieved CMI (AUC = 0.78; 95% CI = 0.64–0.92; *p* = 0.001). (**B**) Calibration plot showing agreement between model-estimated probabilities and observed CMI rates. The Brier score (0.177) indicates acceptable calibration. (**C**) Calibrated probability curve illustrating the relationship between the model-derived score and the estimated probability of achieving CMI. The solid horizontal line denotes the Youden-optimal cutoff (*p* = 0.47; sensitivity 0.75; specificity 0.79). Background color bands indicate low (<0.30), intermediate (0.30–0.70), and high (≥0.70) probability zones.

As summarized in Table 3, all models showed acceptable variance inflation factors (VIF ≤ 1.84), indicating no multicollinearity. Among them, the SBI-based model demonstrated the best fit (AIC = 63.6) and discriminative performance (AUC = 0.78, 95% CI: 0.64–0.92). The SBI model yielded sensitivity, specificity, positive predictive value, and negative predictive value of 75.0%, 79.3%, 71.4%, and 82.1%, respectively. The performance of the SBI-based logistic model was evaluated by both discrimination and calibration analyses (Figure 3). The ROC curve demonstrated good discrimination, with an AUC of 0.78 (95% CI: 0.64–0.92, *p* = 0.001; Figure 3A).

Calibration was acceptable, as indicated by a Brier score of 0.177 (Figure 3B).

Furthermore, the calibrated probability curve (Figure 3C) illustrated a consistent relationship between the model-derived scores and the model-estimated probability of CMI achievement.

## 4. Discussion

In this proof-of-concept study, nocturnal scratching behavior quantified by a wearable device was associated with physician-assessed severity (EASI), serum biomarker levels (TARC), and patient-reported symptoms (POEM) in children with atopic dermatitis. Participants received topical therapy reflecting real-world clinical practice, including maintenance treatment for mild disease and induction therapy for moderate-to-severe disease. Although these treatment strategies differ in intent, the present analysis was not designed to evaluate treatment efficacy per se. Rather, nocturnal scratching metrics were averaged across the monitoring period to examine whether they captured overall disease burden during this interval irrespective of treatment phase. Moreover, changes in scratching metrics paralleled changes in clinical severity over time, supporting their responsiveness as short-term monitoring measures. Building upon these findings, we further explored logistic regression models to assess whether scratch-derived metrics could identify children who achieved clinically meaningful improvement defined by combined physician- and patient-reported outcomes. Among the models examined, the scratch burden index (SBI) showed the most favorable discriminative performance, suggesting that integrated measures of scratching intensity and duration may best reflect clinically relevant change. Taken together, these findings indicate that patient-generated nocturnal scratching data may function as objective digital monitoring indicators of disease activity and treatment-responsive change in pediatric AD.

The observed associations between nocturnal scratching behavior and clinical severity, particularly physician-rated EASI, provide objective support for the well-established itch–scratch cycle in AD [19]. This bidirectional process, in which pruritus induces scratching and scratching aggravates skin inflammation, is considered a central mechanism underlying disease persistence and exacerbation [19]. Consistent with previous actigraphy- and accelerometer-based studies, our findings demonstrate that objectively quantified nocturnal scratching using a consumer device in a real-world home setting is closely linked to clinical disease activity [8,20,21].

Notably, scratching metrics aggregated during the monitoring period also correlated with serum TARC levels, a well-established biomarker of inflammation in AD that reflects both disease severity and treatment responsiveness [16,22]. However, the temporal pattern of these associations warrants careful interpretation. Although our initial hypothesis assumed that scratching behavior during the monitoring period would act as an upstream driver of subsequent disease severity, correlations with EASI were consistently stronger at baseline (visit 1) than at the end of monitoring (visit 2). This pattern suggests that antecedent disease severity may more strongly influence subsequent scratching behavior over a short observation window, rather than scratching serving as a simple unidirectional determinant of later clinical outcomes. A similar temporal ambiguity was observed for serum TARC, which was measured only at visit 2 and showed stronger correlations with baseline EASI than with contemporaneous EASI, limiting inferences regarding directionality. The absence of baseline TARC measurements precludes evaluation of temporal changes in biological disease activity, and these associations should therefore be interpreted as cross-sectional rather than longitudinal. Despite these limitations, aggregated scratching metrics during the monitoring period showed consistent associations with post-treatment EASI, and changes in scratching metrics paralleled changes in EASI. Taken together, these findings indicate that wearable-derived scratching metrics capture multiple dimensions of disease activity, reflecting both underlying inflammatory burden and short-term symptom dynamics, and may therefore serve as complementary monitoring indicators alongside conventional clinical and laboratory measures.

Building on these behavioral insights, we developed an exploratory logistic regression model to distinguish children who achieved clinically meaningful improvement (CMI) in both objective signs and patient-reported outcomes. The model, based on the scratch burden index (SBI) derived from nocturnal scratching during the early phase of treatment, illustrates a potential framework for providing individualized, interpretable feedback in pediatric atopic dermatitis. Rather than delivering automated alerts [23], this approach emphasizes patient understanding and self-assessment, which may support engagement and shared decision-making between patients and clinicians [24]. However, given its exploratory nature, the model requires prospective validation in independent cohorts before any clinical application.

This study has several limitations. First, the exploratory logistic model has not been validated in independent cohorts, and its performance and generalizability should therefore be interpreted with caution. Second, the observational design precludes causal inference regarding the relationship between scratching behavior and disease activity. Third, the single-center setting and modest sample size may limit statistical power and external validity. This also constrains the statistical robustness of the findings, particularly for exploratory analyses and model performance. Fourth, the relatively short monitoring period (1–2 weeks) did not allow evaluation of long-term changes, seasonal variation, or sustained treatment responses. Fifth, patient-reported outcomes such as POEM and NRS were partly collected at home, introducing potential reporting bias and missing data. In addition, topical treatment was not fully standardized, and treatment adherence was not formally assessed, which may have influenced short-term changes in scratching behavior. Sixth, although validated in adults, child-specific validation of accelerometry-derived scratching metrics remains limited. Seventh, serum TARC was measured only at visit 2 to minimize patient burden, which precludes assessment of temporal changes in biological disease activity and limits interpretation to cross-sectional associations. Finally, the scratching metrics were derived from a single consumer device, and further studies are needed to confirm reproducibility across devices and to evaluate feasibility and integration into routine clinical workflows.

In conclusion, this proof-of-concept study showed that nocturnal scratching measured by a wearable device shows moderate associations with baseline disease severity and short-term clinical improvement during topical treatment in children with AD. An exploratory logistic model using scratch-derived metrics demonstrated moderate discriminative performance for clinically meaningful improvement; however, its predictive validity remains to be established and requires external validation in larger cohorts. These findings support the potential utility of objective scratching metrics as complementary, treatment-responsive digital measures. However, given the cross-sectional nature of biomarker analyses, treatment heterogeneity, and device-related limitations, these results should be interpreted with caution, and further prospective validation is required before clinical application.

## Figures and Tables

**Table 1:** Characteristics of the subjects.

Factors		*p* Value
Number of subjects	50	
Sex (male), N (%)	30 (60%)	
Age, median (range), years	9 (5–14)	
Monitoring period, median (range), nights	8 (5–13)	
Sleep time, median (range), hours:minutes	9:10 (6:44–10:44)	
Severity ^#1^ at visit 1, N (%)		
Mild	16 (33%)	
Moderate	26 (53%)	
Severe	7 (14%)	
EASI, median (range)		
Visit 1 (baseline)	11 (1.6–53.3)	<0.0001 ^#2^
Visit 2 (final)	3.7 (0.2–16)	
POEM, median (range)		<0.0001 ^#3^
Visit 1 (baseline)	12 (1–28)	
Midpoint (home)	7 (0–28)	0.0002 ^#3^
Visit 2 (final)	5 (0–17)	<0.0001 ^#4^
CDLQI at visit 2, median (range)	3 (0–11)	
TARC at visit 2, median (range), pg/mL	398 (102–9285)	
Comorbid allergic diseases, N (%)		
Food allergy	23 (46%)	
Asthma	15 (30%)	
Rhinitis	36 (72%)	
Conjunctivitis	15 (30%)	

^#1^ Severity was classified according to published EASI score criteria: Clear (0), Almost Clear (0.1–1.0), Mild (1.1–7.0), Moderate (7.1–21.0), Severe (21.1–50.0), Very Severe (50.1–72.0). In this table, severity is shown for baseline (Visit 1). No patients were in the Clear, Almost Clear, or Very Severe categories. ^#2^ Paired *t* test. ^#3^ Mixed effect model. ^#4^ Dunnett’s multiple comparison test versus baseline. *p* values are shown only for variables with statistical comparison; other variables are descriptive only.

**Table 2:** Linear regression and correlation between changes in scratch metrics and physician-assessed disease severity (EASI).

Predictor	Outcome	Pearson r	95% CI for r	*p*-Value	R^2^
SCR slope	ΔEASI	0.69	0.48–0.80	<0.0001	0.45
SDR slope	ΔEASI	0.71	0.53–0.82	<0.0001	0.50
SBI slope	ΔEASI	0.67	0.48–0.80	<0.0001	0.44

Pearson correlation coefficients (r) between the slopes of scratch metrics and the slope of EASI, SCR; scratch rate (/h), SDR; scratch duration ratio (%), SBI; scratch burden index (m/h), 95% CI; 95% confidence interval, R^2^: coefficients of determination.

**Table 3:** Performance of logistic regression models for distinguishing clinically meaningful improvement using smartwatch-derived scratch metrics.

Model	VIF	AIC	AUC (95% CI)	Cutoff	Sensitivity	Specificity	PPV	NPV
1	1.117	67.0	0.76 (0.62–0.90)	0.39	80.0%	65.5%	61.5%	82.6%
2	1.244	68.0	0.78 (0.64–0.92)	0.45	75.0%	79.3%	71.4%	82.1%
3	1.837	63.6	0.78 (0.64–0.92)	0.47	75.0%	79.3%	71.4%	82.1%

Model 1: Scratch Count Rate (SCR); Model 2: Scratch Duration Ratio (SDR); Model 3: Scratch Burden Index (SBI). Each parameter was represented by its mean and slope during the monitoring period. Clinically meaningful improvement was defined as the concurrent achievement of EASI-50 and a ≥4-point reduction in POEM. AIC: Akaike information criterion; VIF: variance inflation factor; AUC: area under the ROC curve; 95% CI: 95% confidence interval; PPV: positive predictive value; NPV: negative predictive value.

## Data Availability

Data are available from the corresponding author upon reasonable request.

## Supplementary Materials

The following supporting information can be downloaded at: https://www.mdpi.com/article/10.3390/jcm15093380/s1, Figure S1: Associations between wearable-derived nocturnal scratch metrics and patient-reported symptoms assessed by POEM; Figure S2: Associations between patient-reported itch intensity assessed by the numerical rating scale (NRS) and clinical severity or biomarker levels; Table S1: Pearson Correlations between Scratch Metrics and NRS, and CDLQI; Table S2: Comparison of Correlation Coefficients Between TARC and Scratch Metrics Versus EASI, as Tested by Steiger’s Z Test; Table S3: Pearson Correlations Among Scratch Metrics.

## Abbreviations

The following abbreviations are used in this manuscript:

AD	Atopic dermatitis
EASI	Eczema Area and Severity Index
POEM	Patient-Oriented Eczema Measure
CDLQI	Children’s Dermatology Life Quality Index
NRS	Numerical Rating Scale
TARC	Thymus and activation-regulated chemokine
SCR	Scratch Count Rate
SDR	Scratch Duration Ratio
SBI	Scratch Burden Index
AUC	Area under the curve
