# Supplementary material for: Smartwatch-Derived Nocturnal Scratching Metrics Capture Disease Activity and Severity in Pediatric Atopic Dermatitis
Source: J Clin Med. 2026 Apr 28;15(9):3380. doi: 10.3390/jcm15093380 (PMC13164348; doi:10.3390/jcm15093380)
Supplement: Supplementary file 1 [file jcm-15-03380-s001.zip › jcm-4183852-supplementary.pdf]

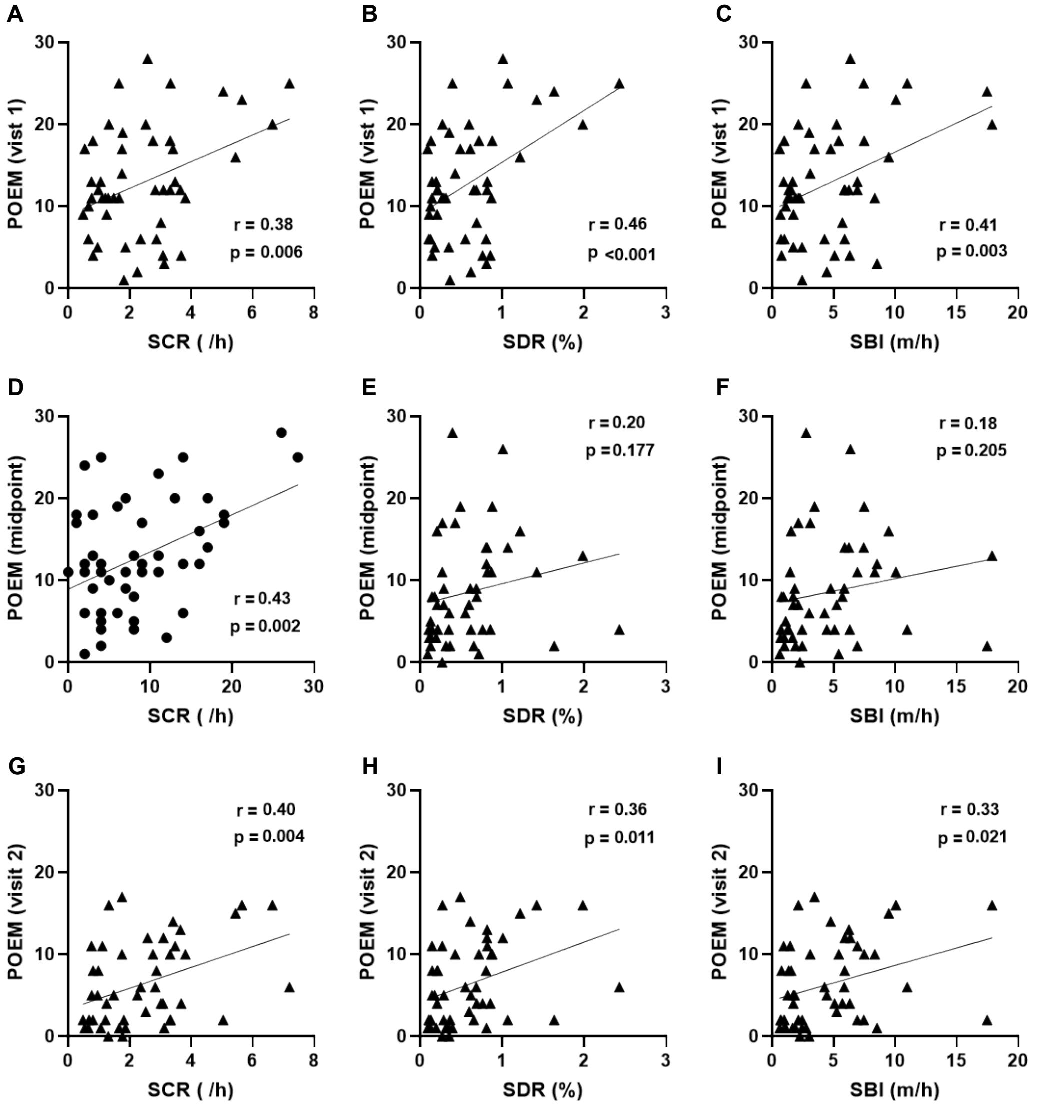

Figure S1.

Associations between wearable-derived nocturnal scratch metrics and patient-reported symptoms assessed by POEM.

Scatter plots show the relationships between scratch count rate (SCR), scratch duration ratio (SDR), and scratch burden index (SBI) with the Patient-Oriented Eczema Measure (POEM) at three time points: visit 1 (baseline; A–C), midpoint during home monitoring (D–F), and visit 2 (final; G–I). Each point represents an individual participant.

Pearson's correlation coefficients (r) and corresponding p-values are shown in each panel. Linear regression lines are displayed for visualization. At baseline and visit 2, all scratch metrics showed modest but statistically significant correlations with POEM. At the midpoint assessment, a significant association was observed for SCR, whereas correlations for SDR and SBI did not reach statistical significance.

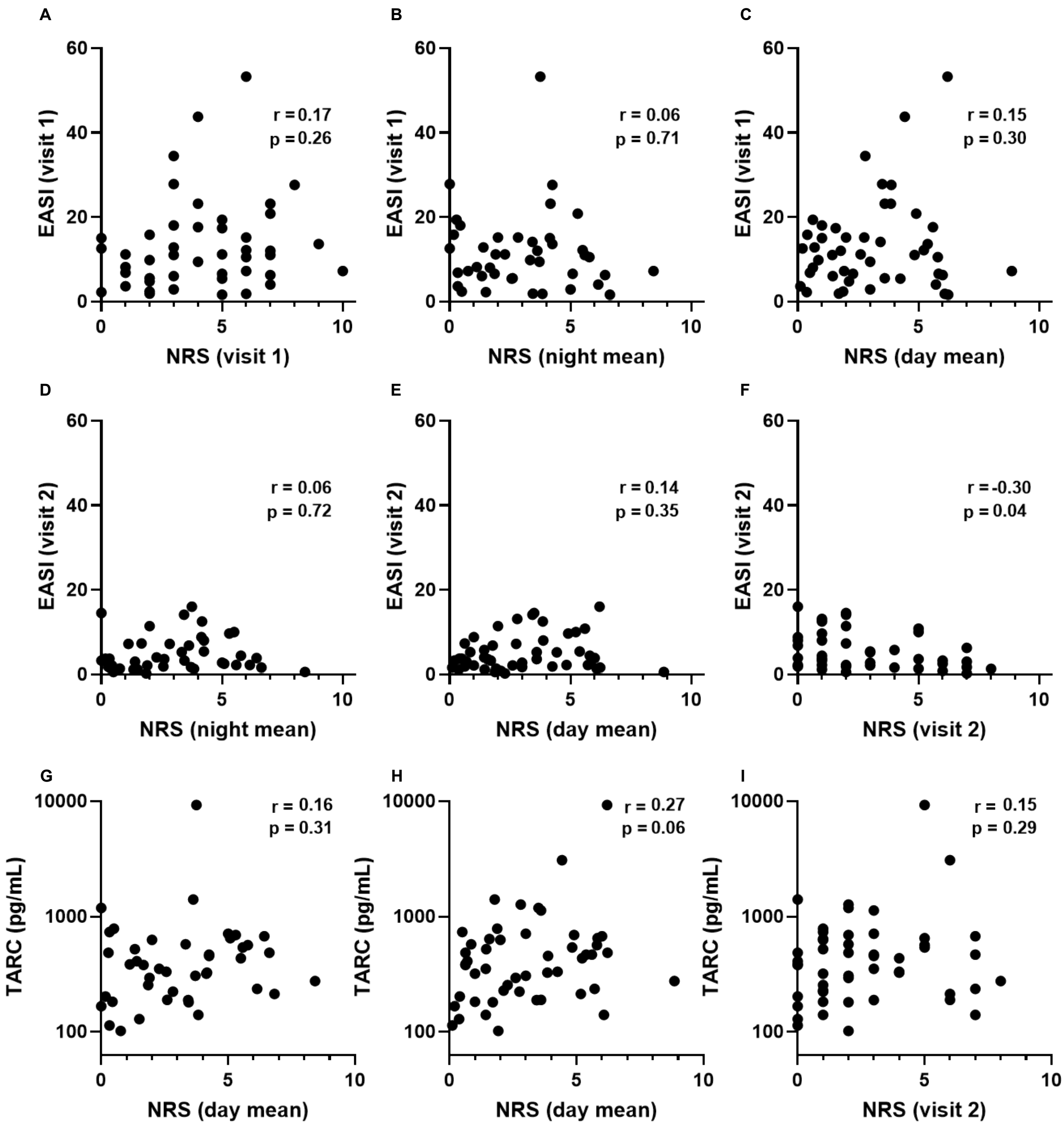

Figure S2.

Associations between patient-reported itch intensity assessed by the numerical rating scale (NRS) and clinical severity or biomarker levels.

Scatter plots show the relationships between NRS scores and physician-assessed disease severity (EASI) at visit 1 (baseline; A–C) and visit 2 (final; D–F), as well as between NRS scores and serum thymus and serum TARC levels measured at visit 2 (G–I). NRS was assessed at visit 1, visit 2, and as mean daytime or nighttime values during the monitoring period, as indicated in each panel. Each point represents an individual participant.

Pearson's correlation coefficients (r) and corresponding p-values are displayed within each panel. Overall, NRS showed weak or no correlations with EASI or serum TARC, with only limited associations reaching statistical significance.

Table S1. Pearson Correlations between Scratch Metrics and NRS, and CDLQI

	SCR	SDR	SBI
NRS at visit 1	0.17 (0.303)	0.15 (0.354)	0.15 (0.375)
Mean daytime NRS	0.22 (0.182)	0.2 (0.225)	0.17 (0.305)
Mean nighttime NRS	0.37 (0.021)	0.37 (0.021)	0.31 (0.061)
NRS at visit 2	0.21 (0.199)	0.23 (0.169)	0.19 (0.255)
CDLQI	0.05 (0.731)	0.05 (0.723)	0.02 (0.892)

Values represent Pearson’s correlation coefficients (r) with corresponding p-values in parentheses. SCR, scratch count rate (episodes/hour); SDR, scratch duration ratio (% of sleep spent scratching); SBI, scratch burden index (m/h)

NRS; numeric rating scale for itch, CDLQI; Children’s Dermatology Life Quality Index

Table S2. Comparison of Correlation Coefficients Between TARC and Scratch Metrics Versus EASI, as Tested by Steiger’s Z Test

Variables	Pearson’s correlation coefficients		
r_1 (TARC vs scratch metric)	0.6 (SCR)	0.58 (SDR)	0.6 (SBI)
r_2 (TARC vs EASI visit 1)		0.68	
r_3 (TARC vs EASI visit 2)		0.45	
Z, r_1 vs r_2 (p value)	–2.84 (0.005)	–3.48 (0.001)	–2.84 (0.005)
Z, r_1 vs r_3 (p value)	4.36 (<0.001)	3.72 (0.001)	4.36 (0.001)
Interpretation	$r_1 < r_2$; $r_1 > r_3$	$r_1 < r_2$; $r_1 > r_3$	$r_1 < r_2$; $r_1 > r_3$
	Weaker than baseline EASI; stronger than follow-up EASI		

r_1 = correlation between TARC and scratch metric; r_2 = correlation between TARC and EASI at baseline (visit 1); r_3 = correlation between TARC and EASI at follow-up (visit 2). Z values from Steiger’s Z test

Table S3. Pearson Correlations Among Scratch Metrics

Pairwise correlations	r (p-value)
SCR (/h) vs SDR (%)	0.97 (<0.001)
SCR (/h) vs SBI (%)	0.92 (<0.001)
SDR (%) vs SBI (m/h)	0.92 (<0.001)

Values represent Pearson’s correlation coefficients (r) with corresponding p-values in parentheses. SCR, scratch count rate (episodes/hour); SDR, scratch duration ratio (% of sleep spent scratching); SBI, scratch burden index (m/h)
